# NMR derived changes of lipoprotein particle concentrations related to impaired fasting glucose, impaired glucose tolerance, or manifest type 2 diabetes mellitus

**DOI:** 10.1186/s12944-023-01801-7

**Published:** 2023-03-24

**Authors:** Tina Kalbitzer, Kristina Lobenhofer, Silke Martin, Markus Beck Erlach, Werner Kremer, Hans Robert Kalbitzer

**Affiliations:** 1 Institute of Biophysics and Physical Biochemistry and Centre of Magnetic Resonance in Chemistry and Biomedicine, University of Regensburg, Universitätsstr. 31, 93040 Regensburg, Germany; 2 Blutspendedienst des Bayerischen Roten Kreuzes Gemeinnützige GmbH, Herzog-Heinrich-Straße 2, 80336 Munich, Germany

**Keywords:** Type 2 diabetes mellitus, Lipoprotein subclasses, NMR spectroscopy, T2D, impaired glucose tolerance, IGT, impaired fasting glucose, IFG, lipoprotein particles, blood donors

## Abstract

**Background:**

Type 2 diabetes mellitus (T2D) and corresponding borderline states, impaired fasting glucose (IFG) and/or glucose tolerance (IGT), are associated with dyslipoproteinemia. It is important to distinguish between factors that cause T2D and that are the direct result of T2D.

**Methods:**

The lipoprotein subclass patterns of blood donors with IFG, IGT, with IFG combined with IGT, and T2D are analyzed by nuclear magnetic resonance (NMR) spectroscopy. The development of lipoprotein patterns with time is investigated by using samples retained for an average period of 6 years. In total 595 blood donors are classified by oral glucose tolerance test (oGTT) and their glycosylated hemoglobin (HbA1c) concentrations. Concentrations of lipoprotein particles of 15 different subclasses are analyzed in the 10,921 NMR spectra recorded under fasting and non-fasting conditions. The subjects are assumed healthy according to the strict regulations for blood donors before performing the oGTT.

**Results:**

Under fasting conditions manifest T2D exhibits a significant concentration increase of the smallest HDL particles (HDL A) combined with a decrease in all other HDL subclasses. In contrast to other studies reviewed in this paper, a general concentration decrease of all LDL particles is observed that is most prominent for the smallest LDL particles (LDL A). Under normal nutritional conditions a large, significant increase of the concentrations of VLDL and chylomicrons is observed for all groups with IFG and/or IGT and most prominently for manifest T2D. As we show it is possible to obtain an estimate of the concentrations of the apolipoproteins Apo-A1, Apo-B100, and Apo-B48 from the NMR data. In the actual study cohort, under fasting conditions the concentrations of the lipoproteins are not increased significantly in T2D, under non-fasting conditions only Apo-B48 increases significantly.

**Conclusion:**

In contrast to other studies, in our cohort of “healthy” blood donors the T2D associated dyslipoproteinemia does not change the total concentrations of the lipoprotein particles produced in the liver under fasting and non-fasting conditions significantly but only their subclass distributions. Compared to the control group, under non-fasting conditions participants with IGT and IFG or T2D show a substantial increase of plasma concentrations of those lipoproteins that are produced in the intestinal tract. The intestinal insulin resistance becomes strongly observable.

## Background

Metabolic syndrome, insulin resistance, and type-2 diabetes are associated with a typical dyslipoproteinemia. Dyslipoproteinemia can be studied elegantly by high resolution ^1^H-NMR spectroscopy as shown initially by the groups of Otvos [1, 2] and Ala-Korpela [3] in this field. An important feature of NMR spectroscopy is the possibility of classifying subgroups of lipoproteins by their size under high-throughput conditions, giving additional information on the size distribution and corresponding particle numbers. Whereas initially NMR analytics was based solely on a chemical shift analysis of the lipoprotein spectra, later the method was improved by additionally using diffusion effects measured by pulsed magnetic field gradients [4, 5]. Since the NMR visibility of different lipoproteins varies strongly in different lipoprotein classes [6], the data evaluation procedure has to be calibrated carefully by the gold standard method analytical ultracentrifugation. However, the latter method is not suitable for large scale studies, therefore, NMR spectroscopy was mainly used in the past for characterizing changes in lipoprotein particle patterns caused by different forms of prediabetes (defined by NIDKK as impaired fasting glucose (IFG) and/or impaired glucose tolerance (IGT)) and manifest type-2 diabetes (T2D) itself [7–14].

In a prospective study by Festa et al. [7] the lipoprotein particle sizes and concentrations were determined by NMR spectroscopy with an average follow-up time of 5.2 years. Increased concentrations of small HDL and large VLDL were positively associated with an increased risk for the development of type-2 diabetes. Another follow-up study of 13 years by Mora et al. [9] showed that increased concentrations of small HDL, small LDL, and large VLDL particles were predictive for a higher risk to develop type-2 diabetes. In a multi-ethnic study of atherosclerosis by Mackey et al. [11], besides changes in lipid concentrations, increased concentrations of VLDL were found to be associated with the development of diabetes mellitus. Wang et al. [10] analyzed the lipoprotein concentrations from male Finnish individuals in native blood serum. It is the only NMR based study where an accurate metabolic classification by oral glucose tolerance test (oGTT) has been performed. The participants were assigned to five classes, non-diabetic participants, participants with impaired fasting glucose (IFG), impaired glucose tolerance (IGT), IFG combined with IGT, and with newly diagnosed type-2 diabetes (T2D). Increased VLDL concentrations were associated with abnormal glucose tolerance as found in IGT and T2D. Decrease of large HDL and increase of small HDL concentrations were consistently observed for individuals with abnormal fasting glucose (IFG and T2D). Sokooti et al. [12] compared the HDL-particle distribution of the post transplantation diabetes mellitus with type-2 diabetes mellitus from other sources. They found the risk to develop type-2 diabetes is decreased, when larger HDL particles prevail. The same was found for the risk to develop T2D for non-transplanted subjects [13]. Tranes et al. [14] studied a small group of lean Chinese with and without insulin resistance and found no differences of the lipoprotein subclass distributions in the two groups. They concluded that mechanistically there is a dissociation between the insulin resistance at the level of glucose metabolism (impaired glucose tolerance) and the dyslipoproteinemia usually described in type 2 diabetes mellitus.

In the present study we focus on a different population, German long time blood donors that are assumed to be healthy according to the rules applying for blood donors. IFG, IGT or manifest T2D were not known for this group before including them in this study. The metabolic state was determined by the “gold standard” oGTT. Based on the oGTT the participants were divided in five metabolic groups, the healthy control group and the four groups with different disorders of the glucose metabolism (impaired fasting glucose, impaired glucose tolerance, umpaired fasting glucose combined with impaired glucose tolerance, manifest type 2 diabetes mellitus). The differences in the lipoprotein particle numbers and concentrations in five different groups were analysed. In addition, we could retrospectively follow the changes in the lipoprotein parameters in retained samples taken at different times in an average period of 6 years before testing.

## Methods

### Study design

The participants of this study were preselected from the pool of blood donors of the Bavarian Red Cross (BRK) by sending the FindRisk questionnaires [15] to 60,000 individuals of this group. 51,021 correctly filled out FindRisk forms were returned. The FindRisk score questionnaire predicts the risk to develop type-2 diabetes within the next 10 years. The concentration of glycosylated hemoglobin HbA1c was determined for 12,773 of these blood donors. On the basis of their FindRisk score and their HbA1c concentration, 4017 persons were invited to the oGTT. Six hundred seventy-one persons accepted the invitation to perform an oral glucose tolerance test (oGTT). Finally, 595 persons fulfilled all criteria for the inclusion in the present diabetes NMR study (Table 1). In addition to the general, quite strict exclusion criteria for blood donors, the following criteria led to an exclusion from the study: (1) previously known perturbations of the glucose metabolism (treated and untreated), (2) medication known to influence the lipid metabolism, (3) failures in the preparation phase of the oGTT. The participants had to adhere to a normal diet rich in carbohydrates (> 150 g/day) at least 3 days prior to testing, a fasting period of 8–11 h prior to testing, and (4) the capillary glucose concentration immediately before oGTT had to be less than 150 mg/mL. The participants were distributed into 5 classes, a control group, groups of individuals with impaired fasting glucose (IFG), with impaired glucose tolerance (IGT), with impaired fasting glucose combined with impaired glucose tolerance (IFG + IGT), and with newly diagnosed type-2 diabetes (T2D). As control group served blood donors with no signs of diabetes. They were selected on the basis of their normal HbA1c concentrations and their normal oGTT values. Fasting EDTA plasma samples before the start of the oGGT test and 120 min after oral administration of 75 g glucose were immediately frozen at -80 C. For most of these candidates, reserve EDTA plasma samples stored at -80 C were available for NMR-spectroscopy from the BIOBANK of the BRK (www.biobank.de). According to the American Diabetes Association (ADA) we define IFG by venous plasma glucose concentrations *c*(*t* = 0) ≥ 100 mg/dL and ≤ 125 mg/dL and *c*(*t* = 120) ≥ 140 mg/dL and < 200 mg/dL, IGT by *c*(*t* = 0) < 100 mg/dL and *c*(*t* = 120) ≥ 140 mg/dL and < 200 mg/dL, T2D by *c*(*t* = 0) > 125 mg/dL or *c*(*t* = 120) ≥ 200 mg/dL, healthy by *c*(*t* = 0) < 100 mg/dL and *c*( *t* = 120) < 140 mg/dL. The majority of Bavarian blood donors have Caucasian ethnicity.

**Table 1:** Description of the study cohort^a^

oGTT group	All (*N* = 595)	Control (* N* = 251)	IFG (* N* = 193)	IGT (* N* = 26)	IFG + IGT (* N* = 68)	T2D (* N* = 57)
Male	380	140	130	18	45	47
Female	215	111	63	8	23	10
Age [years]	54.3 ± 8.9	52.8 ± 10.1	54.6 ± 7.9	57.2 ± 7.7	56 ± 8.1	56.4 ± 7.0
BMI [kg/m^2^]	28.8 ± 4.1	27.9 ± 4.0	29.0 ± 3.8	29.4 ± 3.5	30.3 ± 4.3	30.4 ± 4.3
HbA1c [%]	5.9 ± 0.4	5.8 ± 0.2	5.9 ± 0.3	5.9 ± 0.4	6 ± 0.3	6.3 ± 0.7
FindRisk Score [a.u]	12.5 ± 3.6	11.4 ± 3.8	12.9 ± 3.3	12.7 ± 2.8	14 ± 3.0	13.7 ± 3.4
*c* ( *t* = 0)^a^ [mg/dL]	103.4 ± 13.4	93.8 ± 4.7	106.7 ± 5.6	95.2 ± 3.9	109.9 ± 6.6	130.3 ± 18.8
*c* (*t* = 120)^b^ [mg/dL]	122.1 ± 43.6	101.2 ± 20.6	106.2 ± 20.3	155.3 ± 14.6	163.8 ± 17.2	210.9 ± 61.4

^a^Concentration of glucose in the oGTT at *t* = 0 min

^b^Concentration of glucose in the oGTT at *t* = 120 min

### NMR-spectroscopy and primary data evaluation

After thawing the frozen samples, 400 μL of EDTA-plasma were used for the NMR experiments performed at 600.2 MHz ^1^H-frequency with a Bruker Avance II NMR spectrometer. The samples were used without any addition of reagents. Three spectra were recorded for every sample at 310 K, one 1D NOESY spectrum (pulse program noesygppr1D) with a mixing time of 10 ms and a repetition time of 5.4 s, and two stimulated spin echo spectra (LED, pulse program ledbpgppr2s1d) using different gradient strengths. The total measuring time per sample was 6 min. The NMR spectra were evaluated using an adapted proprietary software (version 2011) from LipoFit GmbH, Regensburg, Germany, as disclosed in our published patents [4, 5]. Fifteen different subclasses of lipoproteins with varying mean diameters *d* were defined in the evaluation program: HDL A, 7.75 nm, HDL B, 9.25 nm, HDL C, 11.5 nm, HDL D, 14.5 nm, LDL A, 17.5 nm, LDL B, 20 nm, LDL C, 21.5 nm, LDL D, 23.5 nm, LDL E, 27.5 nm, IDL, 35 nm, VLDL A, 50 nm, VLDL B, 70 nm, chylomicron remnants (CM Re), 90 nm, small chylomicrons (CM A), 125 nm, and large chylomicrons (CM B) 375 nm. For additional information see Table 3. The intensities were corrected according to Baumstark et al. [6] for visibility effects at 310 K by multiplying the lipoprotein concentrations obtained from the direct integration by 1.00, 1.30, 1.11, 1.19, 1.27, and 1.36, VLDL (including IDL), LDL, HDL A, HDL B, HDL C, and HDL D, respectively. For chylomicrons correction factors have not been published and thus the correction factor was set to 1.0.

The concentrations of Apo-A1, Apo-B100, and Apo-B48 were calculated from the particle concentrations of the corresponding particles determined by NMR. The Apo-A1 concentration was calculated from the total concentrations of the HDL particles, the Apo-B100 concentration from the total concentrations of the LDL and VLDL particles, and the Apo-B48 concentration from the total concentrations of chylomicron particles, assuming a stoichiometry of 2:1, 1:1, and 1:1, respectively. Since the apolipoprotein concentrations are indirectly derived from the NMR particle numbers, the suffix NMR is used for these concentrations in the following.

### Statistical evaluation

Date were evaluated with the SPSS-software package, version 25.0 for windows (IBM) and the R-program, version 3.6.1 (R Core Team, 2019). The Kolmogorov–Smirnov test together with the Lilifors correction was used to test the normal distribution of data. The *t*-test was used to determine the significance of differences between classes when they were sufficiently well normally distributed. Otherwise the non-parametric Kruskal–Wallis H-test and the Mann–Whitney U-test were used. Particle concentrations at different times were fitted to a linear function of time. Time *t* = 0 is the extrapolation to the time of the oGTT test. The result of the oGGT was not included into the fit since it was recorded under fasting conditions.

### Ethical aspects

The ethical aspects of the study were positively reviewed by the ethics commission of the Bayerische Landesärztekammer (#08,055) at July 29, 2008. They abide the Declaration of Helsinki.

## Results

### Description of the study cohort

**Fig. 1:**
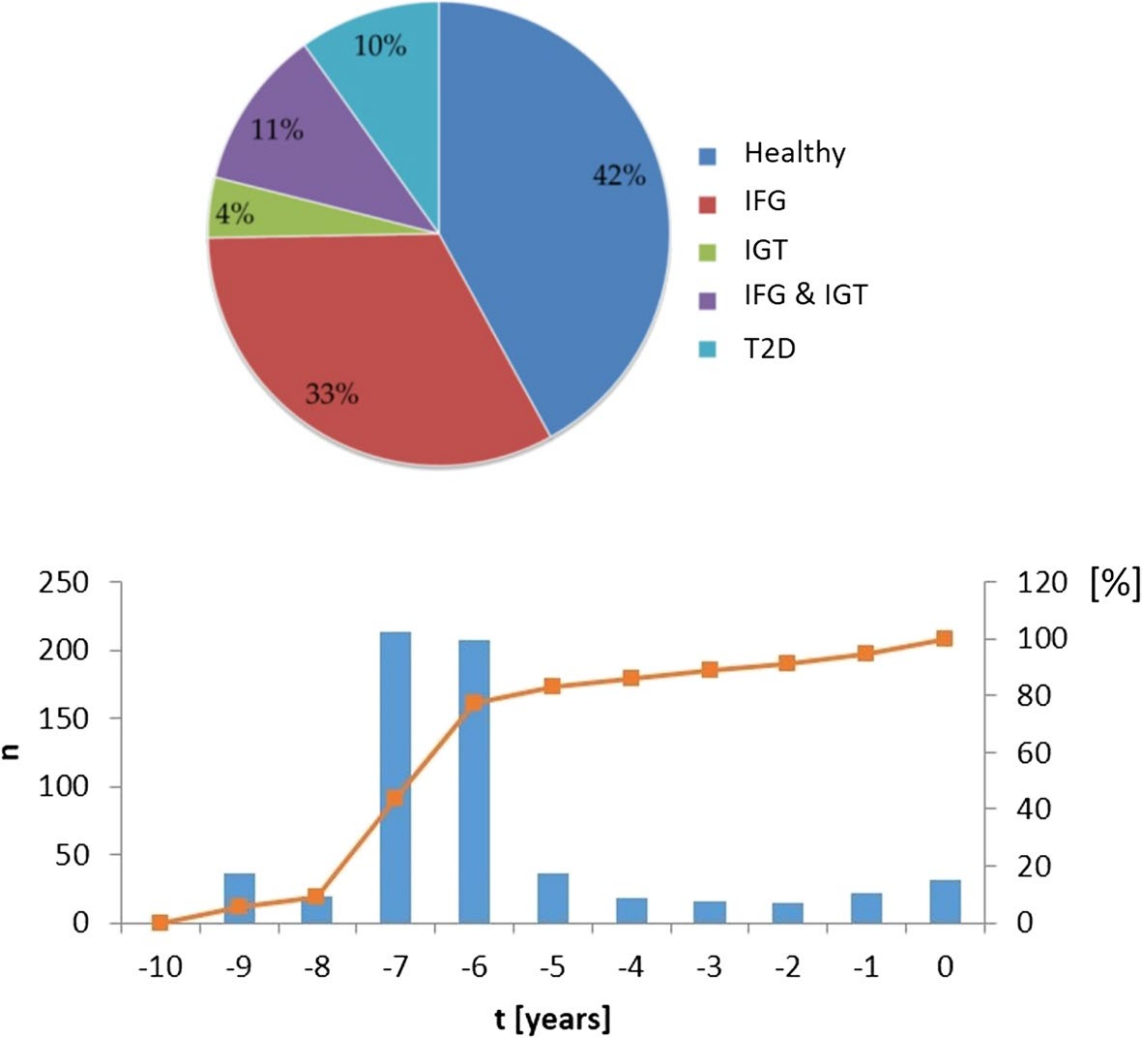
The study cohort. (Top) Distribution of the different oGTT groups in the study. (Bottom) Distribution of the frequency n of whole set of donors that gave the first sample –*t* years before the final oGTT. (Blue) absolute frequency, (orange) cumulative frequency in %

Table 1 summarizes the features of the study cohort, Fig. 1 represents the distribution of the participants to the different groups. The majority of Bavarian blood donors have Caucasian ethnicity. Overall, 215 females and 380 males were accepted in the study. The ratio of 0.56 approximates also the ratio of the two sexes in the cohort of blood donors. The BMIs in all groups are quite similar with an average of 28.8 kg m^−2^, compared to the control group of healthy participants (nonIGT + nonIFG + non2TD), the average value of diabetics is only higher by 9% (Table 1).

All participants were healthy according to the German strict rules for blood donors, diabetes was not known before performing the oGTT tests of the study. Especially, the subjects did not obtain treatment of possible perturbations of their glucose or lipid metabolism. Only persons of age 18 to 68 are accepted as blood donors and thus to the present study. The age distribution was also quite similar in all groups with an average age of 54.3 years and the mean values between the group of heathy subjects and subjects with T2D differ only by 3.5 years. Only small differences between the groups are observed for HbAc1 concentrations and the FindRisk score. Note that subjects with previously known impairment of the glucose metabolism were excluded from the study. In summary, the five groups of the study cohort are quite well matched with respect to age, BMI, and the general risk to develop T2D as predicted by the FindRisk score.

Only for a part of the study participants (all of them were subjected to an oGTT under fasting conditions) suitable NMR samples directly frozen at -80 C at the oGTT were provided for this study (Table 1). That means that for some participants only reserve samples for the lipoprotein analytics by NMR were available. The cohort studied here is part of a larger cohort used to find out the correlation between the FindRisk score and the HbAc1 concentrations [16].

### Estimation of apolipoprotein concentrations by NMR

Generally, it is to be expected that the apolipoproteins directly involved in the recognition of their specific receptor have a fixed stoichiometry for a given type of lipoprotein. There is a good evidence that Apo-A1 is the characteristic apolipoprotein for HDL-particles, Apo-B100 for the non-HDL particles that is LDL and VLDL, and Apo-B48 for the chylomicrons and chylomicron remnants. As consequence, their concentrations should be proportional to the concentrations of their correspondent particles. The proportionality between the NMR derived particle numbers and the apolipoprotein concentrations has already shown experimentally for HDL and non-HDL particles in the serum [17, 18]. However, the exact stoichiometry is still under discussion. We assumed the most likely stoichiometry of 2:1, 1:1, and 1:1 for calculating the apolipoprotein concentrations of Apo-A1, Apo-B100, and Apo-B48 determined by NMR and accordingly call this value Apo-A1_NMR_, Apo-B100_NMR_, and Apo-B48_NMR_ (see also Discussion).

### Lipoprotein particle concentrations related to fasting and normal nutritional conditions

From all individuals of the study cohort oGGT test results were available (Table 1). However, not for all of them plasma samples for NMR analysis were taken during the oGTT test (time *t* = 0) that would reflect the NMR derived lipoprotein state under fasting conditions. For most of the participants reserve samples were taken before *t* = 0, when they donated blood, and were stored in the BioBank at -80 C. For more than 80% of the participants of this study reserve samples older than 6 years were available, with some samples taken at *t* = -10 years (Fig. 1). From all samples NMR spectra were recorded, in total 10,921 sets of plasma NMR spectra were analyzed.

**Fig. 2:**
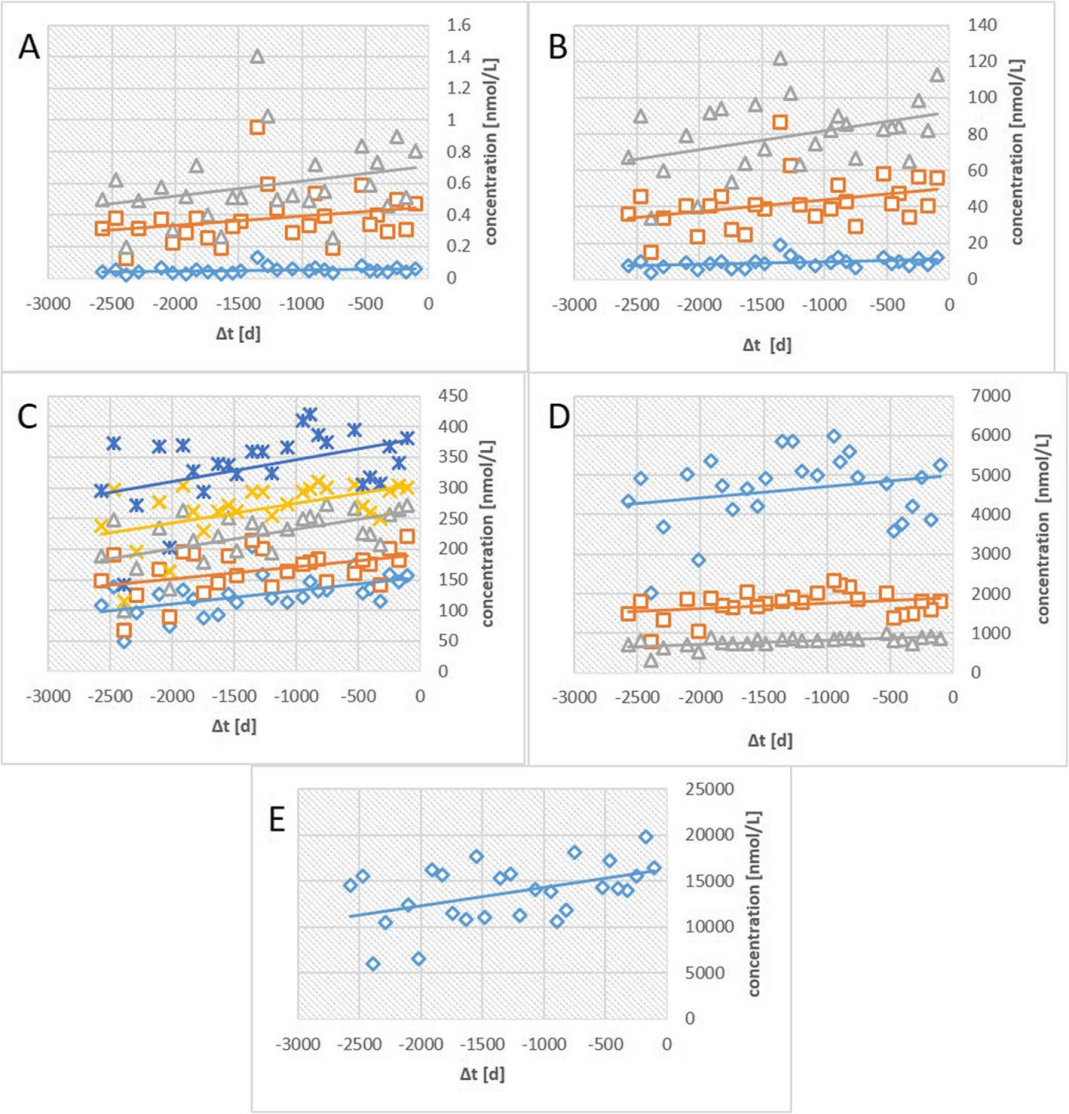
Time dependence of the lipoprotein particle concentrations of an individual with newly diagnosed T2D. Time *t* = 0 is the time of the oGGT. **A** Concentrations of  CM B,  CM A,  CM Re, **B** of  VLDL B,  VLDL A,  IDL, **C** of  LDL E,  LDL D,  LDL C,  LDL B,  LDL A, **D** of  HDL B,  HDL C,  HDL D (**E**) of  HDL A

The time dependence of most of the lipoprotein particle concentrations can be fitted sufficiently well with a linear relation. An example of a blood donor with freshly diagnosed T2D is shown in Fig. 2. The blood donors are asked to eat as every day before blood donation. Of course, their actual NMR lipoprotein profile determined will also depend on the time, when the plasma is taken after food ingestion and the food content itself. Therefore, larger fluctuation of the lipoprotein particles concentrations around the line of best fit are to be expected. The mean changes of particle concentrations (the slopes of the straight lines) are summarized in Table 2. For the control group, for the group with combined IFG and IGT, and for the T2D-group the slope is always positive, meaning that all concentrations increase with time. Only in the groups of participants diagnosed with IFG or IGT, a decrease of concentrations of some lipoproteins with time is observed. Especially, impaired glucose tolerance leads to a decrease of lipoprotein concentrations of almost all particle classes. The relative concentration increases per year are moderate (of the order of 1%). The spread between individuals is significantly larger (data not shown). However, always clear trends are observed with time, allowing to calculate the values expected at time *t* = 0. They represent the average particle concentrations under “normal” nutritional conditions. The values for non-fasting conditions determined by the long-term fit of the data extrapolated to time *t* = 0 are probably more representative of the “normal” non-fasting state of the individuals, since this method decreases variations caused by isolated cases of extensive food consumption.

**Table 2:** Time course of lipoprotein particle concentrations in the years before testing^a^

	**Control**		**IFG**		**IGT**		**IFG + IGT**		**T2D**	
					**<Δ*c*/Δ*t* >**					
	**[nM/y]**	**[%/y]**	**[nM/y]**	**[%/y]**	**[nM/y]**	**[%/y]**	**[nM/y]**	**[%/y]**	**[nM/y]**	**[%/y]**
**HDL A**	103.66	0.61	-17.89	-0.10	178.85	1.06	23.00	0.13	83.22	0.46
**HDL B**	68.26	1.48	88.33	1.96	-98.19	-2.19	138.34	2.89	111.33	2.54
**HDL C**	35.04	1.46	42.71	1.88	-38.69	-1.68	68.26	3.02	46.36	2.22
**HDL D**	12.41	1.08	11.68	1.01	-0.37	-0.03	23.00	1.97	15.70	1.37
**Apo-A1**_**NMR**_	109.68	0.22	62.42	0.12	20.81	0.04	126.29	0.25	128.30	0.25
**LDL A**	5.11	1.10	5.84	1.30	-4.38	-0.95	9.13	2.02	4.75	1.13
**LDL B**	3.65	0.99	2.56	0.70	-2.56	-0.68	6.21	1.67	3.29	0.92
**LDL C**	4.38	1.33	1.83	0.57	-1.83	-0.56	5.11	1.57	1.83	0.59
**LDL D**	1.46	0.65	0.00	0.00	-1.46	-0.64	1.46	0.65	0.37	0.17
**LDL E**	1.83	1.03	0.00	0.00	-2.92	-1.56	2.56	1.33	1.10	0.58
**IDL**	0.37	0.43	-0.37	-0.43	-1.46	-1.69	0.37	0.41	0.00	0.00
**VLDL A**	0.00	0.00	-0.37	-0.79	-1.10	-2.28	0.73	1.48	0.37	0.75
**VLDL B**	0.00	0.00	0.00	0.00	-0.37	-3.40	0.00	0.00	0.00	0.00
**Apo-B100**_**NMR**_	2.10	0.12	1.19	0.07	-2.01	-0.12	3.19	0.19	1.46	0.09
**CM Re**	0.00	0.00	0.00	0.00	0.00	0.00	0.00	0.00	0.00	0.00
**CM A**	0.00	0.00	0.00	0.00	0.00	0.00	0.00	0.00	0.00	0.00
**CM B**	0.00	0.00	0.00	0.00	0.00	0.00	0.00	0.00	0.00	0.00
**Apo-B48**_**NMR**_	0.00	0.00	0.00	0.00	0.00	0.00	0.00	0.00	0.00	0.00

^a^Data are taken from the reserve samples. Note that disorders of the glucose metabolism were newly diagnosed for all participants of this study and that values correspond to normal nutritional conditions. The particle concentrations *c* were linearly fitted as function of the time *t* before the oGTT. <Δ*c*/Δ*t* > is the mean slope of the line of best fit, either expressed in nM/year or %/year. The percents are related to the particle concentrations extrapolated to *t* = 0. Number of participants 595. The Apo-A1_NMR_ concentrations were calculated from the HDL concentrations assuming 2 Apo-A1 molecules /HDL-particle, the Apo-B100_NMR_ concentrations were calculated as the sum of VLDL, IDL and LDL particle concentrations assuming 1 Apo-B100 molecule/particle, the Apo-B48 concentrations were calculated as the sum of all chylomicron particle concentrations assuming 1 Apo-B48 molecule/particle (see Discussion)

Table 3 summarizes the particle concentrations in the five groups under fasting and average nutritional conditions. Fasting has only a small influence on the mean lipoprotein concentrations of the main lipoprotein classes for the control group of healthy volunteers. Even the concentrations of particles related directly to food intake (chylomicrons and chylomicron remnants) are not changed much by fasting in this group. This confirms that the food intake happened usually several hours before blood donation. Only the average volume of these particles is increased after food intake in the healthy control group indicating that more lipids are transported from the guts (Table 4). We observed somewhat larger changes in the subclass patterns of the control group by fasting with the largest relative decrease by -2.2% for the average values of the smallest HDL particles (HDL A) compensated by an increase of larger HDL particles (HDL C and HDL D). Much larger particle concentration changes induced by fasting are clearly observed for subjects with impaired glucose metabolism with the largest effects observed for manifest type 2 diabetes mellitus. It strongly reduces the general concentrations of lipoprotein particles of all classes. The largest changes are observed for the large particles (chylomicrons and VLDL) with a maximum effect found for large chylomicrons (CM B) with an increase by 22.7%. The average volumes and particle concentrations of chylomicrons increase by 6.8% and 19.2% under average nutritional conditions, respectively (Table 3).

**Table 3:** Comparison of lipoproteins concentrations obtained under fasting and non-fasting conditions^a^

		**Control**	**IFG**	**IGT**	**IFG + IGT**	**T2D**
***N***_**f**_		**196**	**167**	**21**	**29**	**19**
***N***_**nf**_		**251**	**193**	**26**	**68**	**57**
**Lipoprotein**	**Fasting state**	***c* [nM]**	***c* [nM]**	***c* [nM]**	***c* [nM]**	***c* [nM]**
**HDL A**	Fasting	16,628 ± 2839	16,885 ± 2939	16,725 ± 2658	17,107 ± 2445	17,408 ± 2776
(7 – 8.5 nm)	Non-fasting	16,963 ± 3320	17,362 ± 2777	16,799 ± 2420	17,285 ± 2251	18,014 ± 2454
	Δ*c*_nf-f_ [%]	2	2.8	0.4	1	3.5
**HDL B**	Fasting	4688 ± 1451	4247 ± 1331	4586 ± 1692	4724 ± 1530	4218 ± 1123
(8.5 – 10 nm)	Non-fasting	4623 ± 1442	4501 ± 1267	4479 ± 1778	4779 ± 1493	4390 ± 1156
	Δ*c*_nf-f_ [%]	-1.4	6	-2.3	1.2	4.1
**HDL C**	Fasting	2445 ± 903	2106 ± 695	2309 ± 1086	2259 ± 612	2004 ± 593
(10 – 13 nm)	Non-fasting	2407 ± 827	2270 ± 749	2300 ± 1049	2263 ± 614	2085 ± 565
	Δ*c*_nf-f_ [%]	-1.6	7.8	-0.4	0.2	4.1
**HDL D**	Fasting	1159 ± 206	1111 ± 170	1184 ± 228	1174 ± 183	1125 ± 147
(13 – 16 nm)	Non-fasting	1154 ± 193	1158 ± 176	1193 ± 235	1170 ± 155	1149 ± 162
	Δ*c*_nf-f_ [%]	-0.5	4.2	0.7	-0.3	2.1
**HDL total**	Fasting	24,921 ± 3488	24,349 ± 3423	24,805 ± 3272	25,265 ± 2840	24,754 ± 3003
(7 – 16 nm)	Non-fasting	25,147 ± 3874	25,290 ± 3234	24,771 ± 3104	25,498 ± 2947	25,638 ± 3128
	Δ*c*_nf-f_ [%]	0.9	3.9	-0.1	0.9	3.6
**Apo-A1**_**NMR**_^b^	Fasting	49,842 ± 6976	48,698 ± 6845	49,611 ± 6543	50,529 ± 5679	49,508 ± 6006
	Non-fasting	50,294 ± 7749	50,581 ± 6468	49,543 ± 6207	50,996 ± 5893	51,276 ± 6255
	Δ*c*_nf-f_ [%]	0.9	3.9	-0.1	0.9	3.6
**LDL A**	Fasting	469.2 ± 127.2	426.4 ± 97.6	469.6 ± 172.6	455.1 ± 81.2	395.1 ± 97.6
(16 – 19 nm)	Non-fasting	465.5 ± 118.5	449.9 ± 105.7	461.4 ± 174.8	452.0 ± 87.9	420.7 ± 86.3
	Δ*c*_nf-f_ [%]	-0.8	5.5	-1.7	-0.7	6.5
**LDL B**	Fasting	370.0 ± 74.3	354.3 ± 61.0	375.2 ± 85.6	374.5 ± 59.8	339.1 ± 59.0
(19 – 21 nm)	Non-fasting	369.4 ± 72.2	367.3 ± 66.0	374.2 ± 89.8	371.1 ± 57.4	358.8 ± 59.1
	Δ*c*_nf-f_ [%]	-0.2	3.7	-0.3	-0.9	5.8
**LDL C**	Fasting	328.5 ± 66.5	314.9 ± 56.4	331.2 ± 78.5	328.6 ± 54.5	296.2 ± 63.4
(21 – 22 nm)	Non-fasting	329.9 ± 70.1	323.0 ± 64.7	326.7 ± 82.9	324.6 ± 53.9	311.5 ± 55.9
	Δ*c*_nf-f_ [%]	0.4	2.6	-1.4	-1.2	5.1
**LDL D**	Fasting	221.3 ± 48.7	216.9 ± 45.1	225.0 ± 54.2	229.0 ± 51.1	205.1 ± 53.7
(22 – 25 nm)	Non-fasting	223.6 ± 57.1	223.1 ± 47.1	227.5 ± 59.1	224.0 ± 47.1	219.2 ± 47.0
	Δ*c*_nf-f_ [%]	1	2.8	1.1	-2.2	6.9
**LDL E**	Fasting	175.6 ± 41.9	180.3 ± 41.0	185.4 ± 35.7	196.6 ± 58.0	169.8 ± 44.1
(25 – 30 nm)	Non-fasting	177.6 ± 53.2	183.2 ± 46.1	187.6 ± 38.2	191.5 ± 45.3	188.8 ± 47.8
	Δ*c*_nf-f_ [%]	1.1	1.6	1.2	-2.6	11.2
**LDL total**	Fasting	1564.7 ± 328.1	1492.9 ± 273.4	1586.4 ± 406.0	1583.7 ± 266.8	1405.2 ± 286.3
(16 – 30 nm)	Non-fasting	1566.0 ± 336.1	1546.5 ± 301.0	1577.2 ± 423.9	1563.1 ± 262.4	1498.8 ± 271.7
	Δ*c*_nf-f_ [%]	0.1	3.6	-0.6	-1.3	6.7
**IDL**	Fasting	83.41 ± 20.95	84.43 ± 20.27	83.41 ± 19.31	91.8 ± 28.37	78.71 ± 23.38
(30 – 40 nm)	Non-fasting	84.87 ± 27.24	85.67 ± 21.48	86.58 ± 19.4	88.13 ± 21.97	86.57 ± 22.98
	Δ*c*_nf-f_ [%]	1.8	1.5	3.8	-4	10
**VLDL A**	Fasting	42.93 ± 13.76	45.08 ± 13.79	45.22 ± 12.18	51.88 ± 23.09	43.17 ± 15.54
(40 – 60 nm)	Non-fasting	43.33 ± 17.74	45.98 ± 14.42	48.03 ± 11.93	49.4 ± 15.7	48.95 ± 16.87
	Δ*c*_nf-f_ [%]	0.9	2	6.2	-4.8	13.4
**VLDL B**	Fasting	9.92 ± 3.22	10.32 ± 3.26	10.1 ± 3.03	12.28 ± 6.02	9.35 ± 3.23
(60 – 80 nm)	Non-fasting	10.06 ± 4.34	10.5 ± 3.42	10.74 ± 2.68	11.35 ± 3.61	11.19 ± 4.11
	Δ*c*_nf-f_ [%]	1.4	1.7	6.3	-7.6	19.7
**IDL and VLDL**	Fasting	136.26 ± 37.08	139.83 ± 36.48	138.72 ± 33.32	155.95 ± 56.55	131.22 ± 40.77
(30 – 80 nm)	Non-fasting	138.27 ± 48.51	142.15 ± 38.65	145.35 ± 32.75	148.88 ± 40.54	146.71 ± 43.34
	Δ*c*_nf-f_ [%]	1.5	1.7	4.8	-4.5	11.8
**Apo-B100**_**NMR**_^b^	Fasting	1700.9 ± 352.0	1632.7 ± 300.9	1725.1 ± 431.3	1739.6 ± 310.7	1536.4 ± 318.3
	Non-fasting	1704.2 ± 372.0	1688.6 ± 329.4	1722.6 ± 449.1	1712.0 ± 294.3	1645.5 ± 306.3
	Δ*c*_nf-f_ [%]	0.2	3.4	-0.1	-1.6	7.1
**CM Re**	Fasting	0.66 ± 0.28	0.7 ± 0.28	0.7 ± 0.25	0.86 ± 0.52	0.68 ± 0.33
(80 – 100 nm)	Non-fasting	0.66 ± 0.35	0.73 ± 0.28	0.77 ± 0.23	0.8 ± 0.33	0.78 ± 0.34
	Δ*c*_nf-f_ [%]	0	4.3	10	-7	14.7
**CM A**	Fasting	0.42 ± 0.17	0.44 ± 0.18	0.43 ± 0.16	0.56 ± 0.35	0.4 ± 0.17
(100 – 150 nm)	Non-fasting	0.42 ± 0.23	0.45 ± 0.18	0.47 ± 0.14	0.51 ± 0.2	0.5 ± 0.23
	Δ*c*_nf-f_ [%]	0	2.3	9.3	-8.9	25
**CM B**	Fasting	0.075 ± 0.039	0.079 ± 0.04	0.076 ± 0.041	0.109 ± 0.083	0.066 ± 0.041
(> 150 nm)	Non-fasting	0.073 ± 0.051	0.082 ± 0.038	0.0810 ± 0.030	0.095 ± 0.046	0.088 ± 0.048
	Δ*c*_nf-f_ [%]	-2.7	3.8	6.6	-12.8	33.3
**CM total**	Fasting	1.15 ± 0.48	1.22 ± 0.49	1.21 ± 0.45	1.53 ± 0.94	1.14 ± 0.54
(80 – 430 nm)	Non-fasting	1.15 ± 0.62	1.26 ± 0.49	1.32 ± 0.38	1.41 ± 0.57	1.37 ± 0.61
	Δ*c*_nf-f_ [%]	0	3.3	9.1	-7.8	20.2
**Apo-B48**_**NMR**_^b^	Fasting	1.15 ± 0.48	1.22 ± 0.49	1.21 ± 0.45	1.53 ± 0.94	1.14 ± 0.54
	Non-fasting	1.15 ± 0.62	1.26 ± 0.49	1.32 ± 0.38	1.41 ± 0.57	1.37 ± 0.61
	Δ*c*_nf-f_ [%]	0	3.3	9.1	-7.8	20.2

^a^For lipoprotein nomenclature see Methods. The values given are the mean ± the standard deviation. The fasting values are from the spectra taken before the oGTT, the non-fasting values were extrapolated to time *t* = 0 from the spectra of reserve samples of the blood donors. Δ*c*_nf-f_ [%], relative particle concentrations c under non-fasting conditions minus those under fasting conditions. Number of participants analysed under fasting conditions (*N*_f_) and non-fasting conditions (*N*_nf_) 432 and 595, respectively. First column, values in bracket represent the diameters assumed for different subclasses

^b^The NMR derived apolipoprotein concentrations were calculated as described in Table 2

**Table 4:** Change of lipoprotein particle diameters and volumes after fasting^a^

	**Fasting state**	**Control**	**IFG**	**IGT**	**IFG + IGT**	**T2D**
***N***_**f**_		**196**	**167**	**21**	**29**	**19**
***N***_**nf**_		**251**	**193**	**26**	**68**	**57**
**Lipoprotein**						
HDL total	Fasting					
(7 – 16 nm)	< *d* > [nm]	8.62	8.55	8.61	8.59	8.52
	< *V* > [nm^3^]	721	701	717	711	693
	Non-Fasting					
	< *d* > [nm]	8.60	8.57	8.60	8.58	8.53
	< *V* > [nm^3^]	715	707	716	709	694
	*Δd*_*nf*-f_ [%]	-0.26	0.23	-0.05	-0.06	0.06
	Δ*V*_nf-f_ [%]	-0.87	0.75	-0.10	-0.25	0.18
	Δ*c*_nf-f_ [%]	**0.9**	**3.9**	-0.1	0.9	**3.6**
LDL total	Fasting					
(16 – 30 nm)	< *d* > [nm]	20.0	20.2	20.1	20.2	20.2
	< *V* > [nm^3^]	9.07E + 3	9.27E + 3	9.14E + 3	9.29E + 3	9.28E + 3
	Non-Fasting					
	< *d* > [nm]	20.0	20.1	20.1	20.1	20.2
	< *V* > [nm^3^]	9.10E + 3	9.20E + 3	9.19E + 3	9.25E + 3	9.35E + 3
	Δ*d*_nf-f_ [%]	0.16	-0.27	0.20	-0.13	0.20
	Δ*V*_nf-f_ [%]	0.31	-0.72	0.57	-0.39	0.74
	Δ*c*_nf-f_ [%]	0.1	**3.6**	-0.6	**-1.30**	**6.7**
IDL and VLDL	Fasting					
(30 – 80 nm)	< *d* > [nm]	42.3	42.4	42.4	42.7	42.4
	< *V* > [nm^3^]	9.06E + 4	9.15E + 4	9.15E + 4	9.38E + 4	9.12E + 4
	Non-Fasting					
	< *d* > [nm]	42.2	42.4	42.5	42.6	42.7
	< *V* > [nm^3^]	9.05E + 4	9.16E + 4	9.22E + 4	9.30E + 4	9.32E + 4
	Δ*d*_nf-f_ [%]	-0.06	0.04	0.25	-0.23	0.62
	Δ*V*_nf-f_ [%]	-0.14	0.11	0.76	-0.87	**2.18**
	Δ*c*_nf-f_ [%]	**1.5**	**1.7**	**4.8**	**-4.5**	**11.8**
CM total	Fasting					
(80 – 430 nm)	< *d* > [nm]	121	121	120	123	119
	< *V* > [nm^3^]	4.55E + 6	4.54E + 6	4.44E + 6	4.88E + 6	4.24E + 6
	Non-Fasting					
	< *d* > [nm]	121	121	120	122	121
	< *V* > [nm^3^]	4.47E + 6	4.54E + 6	4.35E + 6	4.69E + 6	4.52E + 6
	Δ*d*_nf-f_ [%]	-0.36	-0.09	-0.42	-0.94	**1.72**
	Δ*V*_nf-f_ [%]	**-1.84**	0.08	**-2.01**	**-3.99**	**6.75**
	Δ*c*_nf-f_ [%]	0.0	**3.3**	**9.1**	**-7.8**	**20.2**

^a^The fasting values are from the spectra taken before the oGTT, the non-fasting vales were extrapolated to time *t* = 0 from the spectra of reserve samples of the blood donors. < *d* > , average particle diameter and < *V* > average volume calculated with the diameters given in Methods assuming a spherical shape.. Δ*d*_nf-f_, Δ*V*_nf-f_, and Δ*c*_nf-f_, relative differences (in %) of particle diameters, volumes, and concentrations under non-fasting conditions minus those under fasting conditions. *N*_f_ and *N*_nf _, number of participants analysed under fasting or non-fasting conditions, respectively. Differences of mean values between fasting and non-fasting subjects with an error probability ≤ 0.05 are presented in bold letters

Compared to fasting conditions, on average, normal nutritional conditions do not lead to a larger change of plasma apolipoprotein concentrations determined by NMR in the control group, the group without disorders of the glucose metabolism. Here, the concentrations of Apo-A1_NMR_, Apo-B100 _NMR_, and Apo.B48 _NMR_ increase by only 0.9, 0.2, and 0%, respectively (Table 3). In contrast, in T2D the Apo-A1 Apo-B100, and Apo-B48 concentrations are significantly increased under non-fasting conditions by 3.6, 7.1, and 20.2%, respectively (Tables 3 and 5). This probably indicates an increased additional fatty acid synthesis in the liver when the plasma glucose concentration is strongly increased by food intake in T2D. The strongest effect of food intake is again observed in the LDL, VLDL, and chylomicron main classes for components with larger size that can carry larger amounts of lipids. The average volume (related to the absolute concentration of lipids transported) is largely increased (Table 4). The concentration of VLDL of the largest subclass VLDL B is increased by more than 19% during normal food intake compared to the situation observed after fasting (Table 3). Even the number and average size of HDL particles is influenced by fasting in persons with T2D.

**Table 5:** Significance of lipoproteins concentration differences between healthy and (pre)diabetic participants under fasting and non-fasting conditions^a^

**Lipoprotein**	**Fasting state**		**IFG**	**IGT**	**IFG + IGT**	**T2D**
**HDL A**	Fasting	Δ*c*_x-H_ [%]	+ 1.5	+ 0.6	+ 2.9	** + 4.7**
(7 – 8.5 nm)	Non-fasting	Δ*c*_x-H_ [%]	+ 2.4	-1.0	+ 1.9	** + 6.2**
	Fasting	*P*_x-H_	0.253	0.851	0.264	**0.008**
	Non-fasting	*P*_x-H_	0.081	0.890	0.100	**0.002**
**HDL B**	Fasting	Δ*c*_x-H_ [%]	**-9.4**	-2.2	+ 0.8	-10.0
(8.5 – 10 nm)	Non-fasting	Δ*c*_x-H_ [%]	-2.7	-3.1	+ 3.4	-5.0
	Fasting	*P*_x-H_	**0.005**	0.647	0.713	0.146
	Non-fasting	*P*_x-H_	0.494	0.514	0.364	0.258
**HDL C**	Fasting	Δ*c*_x-H_ [%]	**-13.9**	-5.6	-7.6	**-18.0**
(10 – 13 nm)	Non-fasting	Δ*c*_x-H_ [%]	**-5.7**	-4.5	-6.0	**-13.4**
	Fasting	*P*_x-H_	** < 0.005**	0.231	0.432	**0.005**
	Non-fasting	*P*_x-H_	0.041	0.343	0.344	**0.005**
**HDL D**	Fasting	Δ*c*_x-H_ [%]	**-4.1**	+ 2.2	+ 1.3	-3.0
(13 – 16 nm)	Non-fasting	Δ*c*_x-H_ [%]	+ 0.3	+ 3.4	+ 1.5	-0.4
	Fasting	*P*_x-H_	**0.022**	0.972	0.876	0.460
	Non-fasting	*P*_x-H_	0.615	0.249	0.205	0.827
**HDL total**	Fasting	Δ*c*_x-H_ [%]	-2.3	-0.5	+ 1.4	-0.7
(7 – 16 nm)	Non-fasting	Δ*c*_x-H_ [%]	+ 0.6	-1.5	+ 1.4	2.0
**Apo-A1**_**NMR**_^b^	Fasting	*P*_x-H_	0.100	0.782	0.683	0.861
	Non-fasting	*P*_x-H_	0.528	0.813	0.279	0.154
**LDL A**	Fasting	Δ*c*_x-H_ [%]	**-9.1**	+ 0.1	-3.0	**-15.8**
(16 – 19 nm)	Non-fasting	Δ*c*_x-H_ [%]	-3.4	-0.9	-2.9	**-9.6**
	Fasting	*P*_x-H_	**0.001**	0.496	0.702	**0.008**
	Non-fasting	*P*_x-H_	0.163	0.437	0.653	**0.015**
**LDL B**	Fasting	Δ*c*_x-H_ [%]	-4.2	+ 1.4	+ 1.2	-8.4
(19 – 21 nm)	Non-fasting	Δ*c*_x-H_ [%]	-0.6	+ 1.3	+ 0.4	-2.9
	Fasting	*P*_x-H_	0.051	0.846	0.820	0.081
	Non-fasting	*P*_x-H_	0.997	0.771	0.520	0.437
**LDL C**	Fasting	Δ*c*_x-H_ [%]	-4.1	+ 0.8	0.0	**-9.8**
(21 – 22 nm)	Non-fasting	Δ*c*_x-H_ [%]	-2.1	-1.0	-1.6	-5.6
	Fasting	*P*_x-H_	0.051	0.729	0.805	**0.031**
	Non-fasting	*P*_x-H_	0.442	0.694	0.800	0.081
**LDL D**	Fasting	Δ*c*_x-H_ [%]	-2.0	+ 1.7	+ 3.4	-7.4
(22 – 25 nm)	Non-fasting	Δ*c*_x-H_ [%]	-0.2	+ 1.8	+ 0.2	-2.0
	Fasting	*P*_x-H_	0.495	0.922	0.709	0.176
	Non-fasting	*P*_x-H_	0.752	0.655	0.555	0.696
**LDL E**	Fasting	Δ*c*_x-H_ [%]	+ 2.7	+ 5.6	** + 11.9**	-3.3
(25 – 30 nm)	Non-fasting	Δ*c*_x-H_ [%]	+ 3.1	+ 5.6	** + 7.8**	+ 6.3
	Fasting	*P*_x-H_	0.217	0.220	**0.0132**	0.653
	Non-fasting	*P*_x-H_	0.074	0.156	**0.012**	0.083
**LDL total**	Fasting	Δ*c*_x-H_ [%]	**-4.6**	+ 1.4	+ 1.2	**-10.2**
(16 – 30 nm)	Non-fasting	Δ*c*_x-H_ [%]	-1.2	+ 0.7	-0.2	-4.3
	Fasting	*P*_x-H_	**0.041**	0.776	0.854	**0.032**
	Non-fasting	*P*_x-H_	0.803	0.994	0.694	0.230
**IDL**	Fasting	Δ*c*_x-H_ [%]	+ 1.2	0.0	+ 10.1	-5.6
(30 – 40 nm)	Non-fasting	Δ*c*_x-H_ [%]	+ 0.9	+ 2.0	+ 3.8	+ 2.0
	Fasting	*P*_x-H_	0.545	0.916	0.202	0.309
	Non-fasting	*P*_x-H_	0.395	0.556	0.136	0.546
**VLDL A**	Fasting	Δ*c*_x-H_ [%]	+ 5.0	+ 5.3	+ 20.8	+ 0.6
(40 – 60 nm)	Non-fasting	Δ*c*_x-H_ [%]	** + 6.1**	** + 10.8**	** + 14.0**	** + 13.0**
	Fasting	*P*_x-H_	0.099	0.364	0.053	0.852
	Non-fasting	*P*_x-H_	**0.026**	**0.049**	**0.003**	**0.020**
**VLDL B**	Fasting	Δ*c*_x-H_ [%]	+ 4.0	+ 1.8	** + 23.8**	-5.7
(60 – 80 nm)	Non-fasting	Δ*c*_x-H_ [%]	+ 4.4	+ 6.8	** + 12.8**	+ 11.2
	Fasting	*P*_x-H_	0.205	0.819	**0.032**	0.417
	Non-fasting	*P*_x-H_	0.096	0.156	**0.006**	0.055
**IDL and VLDL**	Fasting	Δ*c*_x-H_ [%]	+ 2.6	+ 1.8	+ 14.5	-3.7
(30 – 80 nm)	Non-fasting	Δ*c*_x-H_ [%]	+ 2.8	+ 5.1	** + 7.7**	+ 6.1
	Fasting	*P*_x-H_	0.248	0.734	0.103	0.629
	Non-fasting	*P*_x-H_	0.160	0.240	**0.029**	0.180
**Apo-B100**_**NMR**_^b^	Fasting	Δ*c*_x-H_ [%]	-4.0	+ 1.4	+ 2.3	-9.7
	Non-fasting	Δ*c*_x-H_ [%]	-0.9	+ 1.1	+ 0.5	-3.4
	Fasting	*P*_x-H_	0.127	0.849	0.667	0.071
	Non-fasting	P_x-H_	0.954	0.881	0.554	0.360
**CM Re**	Fasting	Δ*c*_x-H_ [%]	+ 6.1	+ 6.1	** + 30.3**	+ 3.0
(80 – 100 nm)	Non-fasting	Δ*c*_x-H_ [%]	** + 10.6**	** + 16.7**	** + 21.2**	** + 18.2**
	Fasting	*P*_x-H_	0.062	0.443	**0.034**	0.927
	Non-fasting	*P*_x-H_	**0.004**	**0.016**	** < 0.001**	**0.011**
**CM A**	Fasting	Δ*c*_x-H_ [%]	+ 4.8	+ 2.4	** + 33.3**	-4.8
(100 – 150 nm)	Non-fasting	Δ*c*_x-H_ [%]	** + 7.1**	** + 11.9**	** + 21.4**	** + 19.0**
	Fasting	*P*_x-H_	0.122	0.564	**0.011**	0.588
	Non-fasting	*P*_x-H_	**0.016**	**0.041**	** < 0.001**	**0.016**
**CM B**	Fasting	Δ*c*_x-H_ [%]	+ 5.3	+ 1.3	+ 45.3	-12.0
(> 150 nm)	Non-fasting	Δ*c*_x-H_ [%]	** + 12.3**	+ 11.0	** + 30.1**	** + 20.5**
	Fasting	*P*_x-H_	0.205	0.936	**0.012**	0.284
	Non-fasting	*P*_x-H_	**0.011**	0.154	** < 0.001**	**0.048**
**CM total**	Fasting	Δ*c*_x-H_ [%]	+ 6.1	+ 5.2	** + 33.0**	-0.9
(80 – 430 nm)	Non-fasting	Δ*c*_x-H_ [%]	** + 9.6**	** + 14.8**	** + 22.6**	** + 19.1**
**Apo-B48**_**NMR**_^b^	Fasting	*P*_x-H_	0.083	0.546	**0.020**	0.804
	Non-fasting	*P*_x-H_	**0.006**	**0.022**	** < 0.001**	**0.012**

^a^Δ*c*_x-H_ represents the relative particle concentrations c in the groups of (pre)diabetic participants (x) minus those in the control group of healthy participants (H). The error probabilities *P* are obtained by using the non-parametric Mann–Whitney U-test. They compare the particle concentrations of the healthy control group with the different (pre)diabetic cohorts. Δ*c* values with an error probability *P*_x-H_ ≤ 0.05 are presented in bold letters. First column, values in bracket represent the diameter ranges assumed for different subclasses. For more details see Table 3

^b^The NMR derived apolipoprotein concentrations were calculated as described in Table 2

A pattern of diet dependent lipoprotein changes similar to T2D is observed for all groups with impaired fasting glucose or impaired glucose tolerance. The only difference is that the lipoprotein concentration changes are smaller. Interestingly, impaired fasting glucose is more similar to T2D concerning the concentration changes of lipoproteins that carry lipids synthesized in the liver (HDL, LDL, IDL, VLDL). In contrast, subjects with impaired glucose tolerance mainly show an increase of lipoproteins synthesized in the intestinal system (chylomicrons). This may indicate differences in the pathophysiology of the two forms of prediabetes (IFG, IGT). Surprisingly, the group characterized by IFG + IGT shows a very different response to fasting than the other groups. Here, fasting leads to a statistically significant increase (not decrease!) of LDL/VLDL type as well as chylomicron particles, in contrast to the changes observed in IFG, IGT, and T2D but particles with some similarities to the control group of healthy subjects.

### Differences in lipoprotein particle concentrations in subjects with and without disorder of glucose metabolism

The differences of lipoprotein particle concentrations between healthy people and people with isolated IFG, isolated IGT, IFG combined with IGT, or manifest T2D are summarized in Tables 3 and 5. These differences can be a consequence of the perturbation of the glucose metabolism but can also represent the consequence of a risk factor associated with a general dyslipoproteinemia. However, a reasonable hypothesis is that they represent lipoprotein concentration changes that mainly are the consequence of the actual metabolic state.

People with impaired fasting glucose (IFG) have an increased number of chylomicrons and VLDL particles compared to the control group under fasting and normal nutritional conditions. The increase of concentrations is about twice as large under non-fasting conditions and becomes statistically significant for all chylomicron subclasses (CM B, CM A, CM Re). The highest increase by more than 12% is observed for the largest chylomicrons. A smaller increase is also observed for VLDL that is with 6.1% significant for VLDL A under non-fasting conditions. IDL concentrations are barely influenced. The total LDL particle number is decreasing relative to the control group with a stronger decrease observable under fasting conditions. The strongest decrease is observed for the smallest LDL particles (LDL A) with -9.1%. However, the decrease of particle numbers is not uniform in all LDL subclasses, in fact, the particle concentration of the largest LDL particles (LDL E) has the tendency to increase (not statistical significant). For HDL, in total a small decrease of particle numbers relatively to the control group is observed with a stronger effect under fasting conditions. Again, the magnitude and sign of these effects vary from subclass to subclass. Statistically significant are the decrease of particle numbers in the HDL B subclass under fasting conditions and the HDL C subclass for fasting and non-fasting conditions (Table 5).

Qualitatively, the same lipoprotein subclass patterns are observable for IFG and T2D. Quantitatively, in cases, that are significant in both groups, the increase relative to the control group is also much stronger (approximately twice) in T2D compared to IFG. This would suggest that the insulin resistance and/or concomitant increased blood glucose are the common factor influencing the lipoprotein profile.

As in IFG and T2D, impaired glucose tolerance leads to strong concentration increases of large lipoprotein particles (chylomicrons and VLDL) compared with the control groups under fasting and normal nutrition conditions. In general, the concentration increase of these particles is larger than in IFG but smaller than in T2D. Under non-fasting conditions the Apo-48_NMR_ concentration is significantly increased by almost 15% (Table 5). Again IGT shows quite small effects on the HDL and LDL subclass concentrations. The only differences concern HDL B and HDL C concentrations under fasting conditions that are strongly reduced in IFG but not in IGT.

Under non-fasting conditions the IFG + IGT group with impaired fasting glucose combined with impaired glucose tolerance shows much larger increases of particles concentrations of most of the subclasses larger particles (VLDL including IDL and chylomicrons) than the groups with isolated IFG or IGT. The particle number changes in the different subgroups in IFG and IGT do not simply sum up to the values found in the IFG + IGT group. Only for the largest particles (VLDL and chylomicrons) this seems to be the case under fasting and non-fasting conditions. Under non-fasting conditions, the increase of large particle concentrations in the IFG + IGT group corresponds rather well to that observed in the T2D-group. Under fasting conditions, the concentration changes observed differ clearly from those observed for T2D. For large chylomicrons (CM B), an increase by + 45.3% is calculated for IFG + IGT, whereas for T2D a decrease by -9.3% is observed (Table 5). Unfortunately, the number of test persons of the IFG + IGT group was too low to reach an error probability *P* < 0.05, meaning that this may also be a statistical error.

It would also be interesting to see, if people without disorders of the glucose metabolism shows differences in their lipoprotein patterns compared to the whole cohort of subjects with IFG, IGT, or T2D. If this cohort behaves like a homogeneous group, the statistical significance should also increase because of the larger number of participants. Under fasting conditions, the changes found to be significant for T2D alone (decrease of the particle numbers of HDL C and LDL A) remain significant for the whole group. The strong increase of HDL A particle numbers is found to be specific for T2D. In addition, an increase of the particle numbers of chylomicron remnants (*P* < 0.004), and a decrease of the particle numbers of HDL B (*P* = 0.007), HDL C (*P* = 0.001), LDL A (*P* = 0.001), LDL B (*P* = 0.04), and LDL C (*P* = 0.03) get significant for the whole group. Under non-fasting conditions, the increase of VLDL A, and of all chylomicron particles numbers becomes even more significant (*P* < 0.003). The decrease of HDL C and the increase of LDL E and VLDL B observed also for T2D gets now significant with the data of the whole group (*P* = 0.027, 0.006, 0.006, respectively).

## Discussion

### The study cohort

It is a general problem that the results of studies primarily reflect statistical properties of the specific cohort of individuals studied. As already mentioned above, the ratio of 0.58 of the two sexes in our study approximates also the ratio of the two sexes in the complete cohort of blood donors of the BRK. The five groups of the study cohort including the control group were matched with respect to age, BMI, and the general risk to develop T2D as shown by the FindRisk score.

Our study cohort consists of long-term blood donors that are healthy according to the strict rules defined for blood donors by the Bavarian Red Cross. A disorder of the glucose metabolism was unknown to the individuals, before they became recruited to the study. It also means that they did not have severe metabolic symptoms leading to a consultation of a medical doctor and thus leading to a specific treatment with antidiabetics. IFG, IGT, or T2D were newly diagnosed by oGTT. In line with this, the mean HbAc1-value of the control group was with 5.8 not much lower than 6.3 of the T2D group. For most subjects with T2D the relative HbAc1-concentration was below the limit of 6.5%, traditionally used for diagnosing T2D. This indicates that in these subjects T2D with increased glucose concentrations had probably prevailed for a relatively short period of time, in agreement with the accession criteria to the study. They were chosen with the aim to identify persons that just developed their disorder of glucose metabolism.

This apparently short history of impaired glucose metabolism may also influence the actually observed apolipoprotein concentrations in T2D. Therefore, the metabolic changes described in this study may better characterize the effect of “pure” insulin resistance of diabetes on the lipoprotein profile and not other concomitant metabolic changes that are known to be partly causative for the development of T2D as the metabolic syndrome. Such a dissociation of the insulin resistance per se and the usually observed dyslipoproteinemia was also described for a cohort of lean Chinese subjects recently [14].

The lipoprotein concentrations determined from the reserve samples change only moderately with time (Table 2). The particle concentrations in all particle classes increased with time for the healthy control group, the group with combined IFG and IGT, and the T2D group. This is to be expected since the BMI and the coupled plasma lipid concentrations usually increase with time (age). However, the mean increase of particle numbers and of apolipoprotein concentrations derived by NMR increased only by 0.25 and 0.12%/year for Apo-A1 and Apo-B100, respectively. Surprisingly, the group with isolated IGT showed a different trend. The particle concentrations of almost all particle groups decreased with time. The only exception were small HDL particles (HDL A) whose concentration increased with time. The reason for this difference is not clear for us but may give information about the pathomechanism of IGT. It would be interesting to study that in more detail.

### Determination of lipoprotein particle and apolipoprotein concentrations by NMR

Initially, only the intensities of NMR lines separated by size dependent chemical shifts and sometimes (as in our case) the size dependent diffusion constants could be used to count the number of lipid protons in the different lipoprotein subclasses. Together with the approximate lipid composition from these line intensities, the particle concentrations were derived. Baumstark et al. [6] showed that a substantial part of the lipid signals are NMR invisible and thus may introduce large errors in the particle concentration determination. With the data from [6] we corrected our concentrations dependent on the specific subclass under consideration and the experimental temperature during data recording (see Methods). Actually, more and more groups realize that apolipoprotein concentrations determined by alternative methods such as immunoassays can be used as independent check of the lipoprotein particle numbers obtained by NMR spectroscopy.

An easily conceivable idea is that vice versa correct particle numbers determined e. g. by NMR can be used to estimate the corresponding apolipoprotein. It is now established in the lipidomics community that the apolipoprotein stoichiometry is fixed in the different main classes of proteins. Chylomicrons and chylomicron remnants contain just one Apo-B48 [19, 20], LDL, IDL, and VLDL on Apo-B100 [17, 21]. Molecular evidence shows that most probably 2 Apo-A1-molecules [22–27] are arranged in an antiparallel manner for stabilizing ordered phospholipid membranes. Based on this fact, recombinant Apo-A1 is used routinely since more than a decade to produce artificial nanodiscs for x-ray and NMR structural studies. These nanodiscs form spontaneously in the presence of lipids and Apo-A1. In some older publications, also more than two ApoA-I are assumed to be bound to large HDL particles [25]. The most likely stoichiometry for Apo-A1 is 2 apolipoproteins per particles. The proportionality of the Apo-A1 and Apo-B100 concentration to the corresponding particle concentrations is experimentally well-established [17, 18]. Accepting this stoichiometry, the concentrations of these apolipoproteins can be approximated by summing up the particle concentrations in the different classes. Since a final verification of the exact stoichiometry is still missing, we annotate the apolipoprotein concentrations determined by NMR with the suffix “NMR” in the tables.

Under fasting conditions we obtain for the control group of subjects without disorders of glucose metabolism using the above stoichiometry Apo-A1 and Apo-B100, concentrations of 49.9 μM and 1.74 μM, respectively. These values obtained from our visibility corrected particle numbers for Apo-A1 and Apo-B100 are quite close to the average values of 51.9 μM and 1.70 μM reported for their control group by Monsoni-Centelles [18] by apolipoprotein specific immunoassays. This consistency check strongly supports the quantitative validity of our NMR analysis. The concentrations of Apo-B48 determined here for the control group are with 1.16 nM substantially smaller than 8.4 nM and 18.5 nM determined by immunoassays and reported by Masuda et al. [19] and Tian et al. [28], respectively.

The determination of the chylomicron particle concentrations by NMR is more tricky, since NMR can distinguish particles on the basis of their size only. Because of the overlap in size of very large VLDL particles with small chylomicrons and chylomicron remnants (see e. g. [29, 30]) these groups can only be partly separated by NMR. This means that the particle numbers of large VLDL B and smaller chylomicrons can only be approximated by using a suitable size cutoff. In addition, weighting factors for the visibility of chylomicrons are not published yet. Because of lack of information we set the weighting factor to 1 in the calculations of chylomicron particle concentrations.

**Fig. 3:**
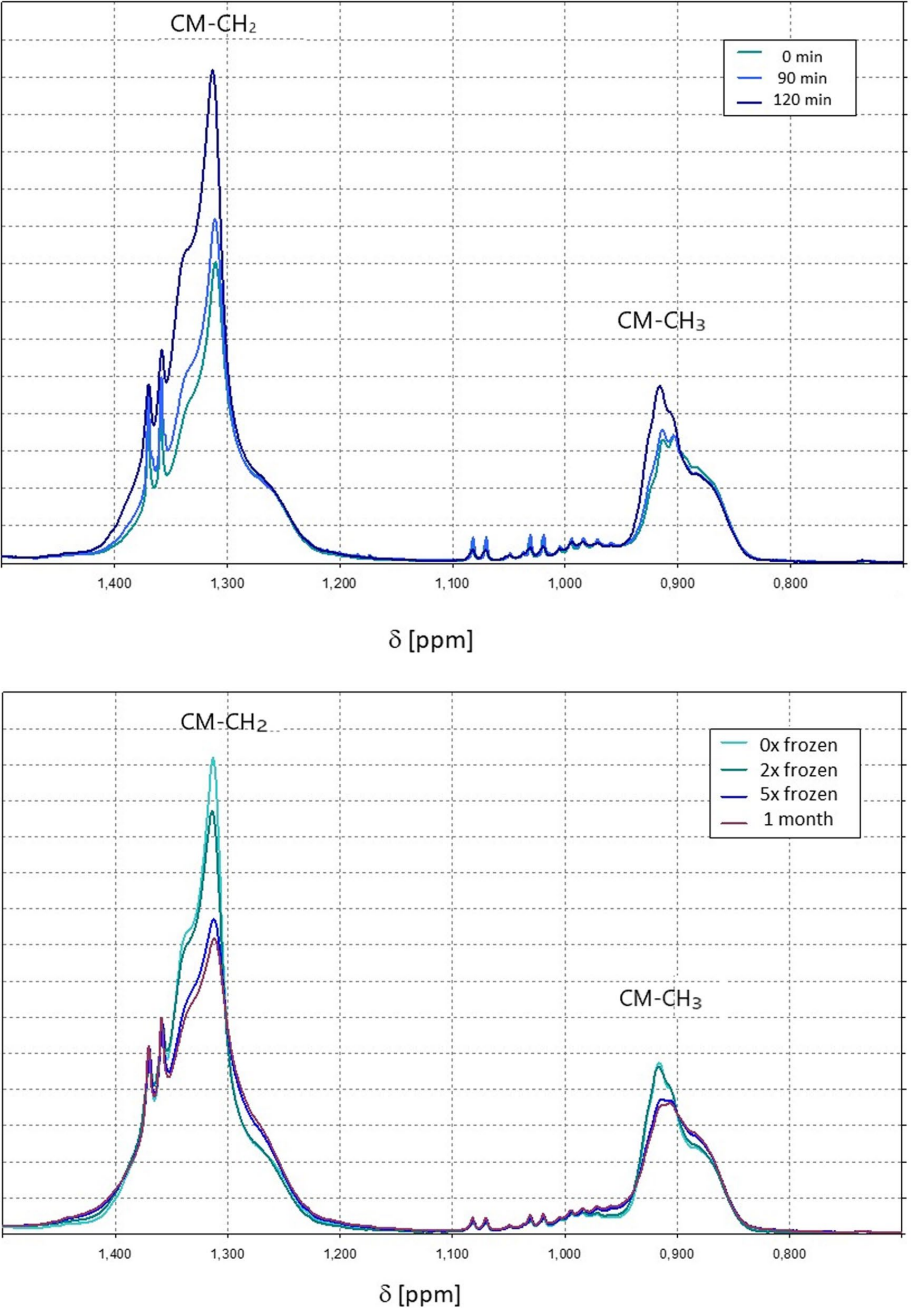
^1^H spectra showing effects of food intake and sample freezing on the methyl and methylene signals of chylomicrons. (Top) Changes of the CH_2_ (CM- CH_2_) and CH_3_ (CM- CH_3_) signals of chylomicrons recorded at different times after food intake. (Bottom) Changes of the CH_2_ (CM- CH_2_) and CH_3_ (CM- CH_3_) signals of chylomicrons recorded after repeated freezing and thawing of the samples (0-times, 2-times, 5-times, 6-times and stored at 253 K). Note that the signals of smaller chylomicrons may overlap with signals of very large VLDLs

Another factor possibly reducing the measured fraction of chylomicrons may be the freezing of samples before NMR analysis. More generally, freezing and (long-term) storage may influence the outcome of the actual measurements under fasting conditions as well as the spectroscopy of the reserve samples. Storage of frozen biological samples at -80 C is assumed to preserve very well their integrity. This is the basis of all biological data bases that intend to provide long-term samples. The only typical effects are sometimes very slow oxidation processes depending on details of the composition of the samples. In general, the critical point is freezing and thawing for complex samples (see e.g. [6]). Figure 3 (top) shows an example for the increase of the chylomicron specific NMR signals (maybe partly superposed by signals of large VLDL) of the corresponding methyl and methylene groups after an intake of a fat rich diet. Figure 3 (bottom) shows the effect of freezing and thawing cycles of this sample. After two cycles the chylomicron NMR signal is only slightly reduced. However, our samples are only frozen once and a signal reduction less than 5% is to be expected (Fig. 3). After several freezing and thawing cycles a stronger reduction of the signal is observed meaning that the chylomicron particle structure is partly destroyed. Note that the NMR signal of LDL and HDL is still unchanged after 6 cycles. There is ample evidence that long-term storage at -80 C or only -20 C does not have an effect on the lipoprotein analysis by NMR (see e. g. [31–34]).

**Fig. 4:**
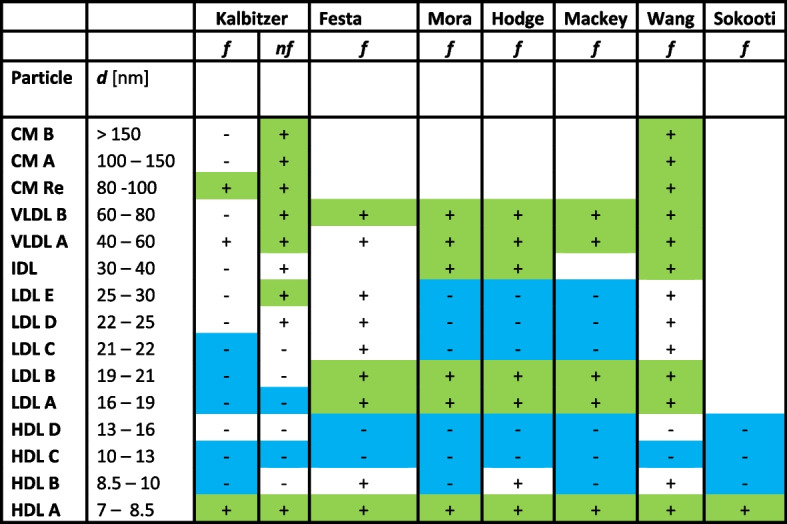
Changes of lipoprotein particle concentrations by impairment of glucose metabolism as described in literature. Concentration increase ( +) or decrease (-) in with IFG, IGT, and T2D, green, significant increase, blue significant decrease. Note that. *f*, fasting, *nf*, non-fasting. The nomenclature of the lipoprotein subclasses and the correspondent particle diameters *d* were similar to those given by Huber et al. [4] and Kaess et al. [35]. The definitions of subclasses and particle diameters *d* vary from study to study. The results of these studies were assigned as good as possible by the diameters to the subclasses given here. Data are taken from Kalbitzer (this study); Festa et al., [7]: Mora et al. [9]; Hodge et al. [8]); Mackey et al. [11]; Wang et al. [10]; Sokooti et al. [13]

Taking as comparison the particle numbers from published NMR based diabetes studies presented in Fig. 4, one obtains Apo-AI concentrations in the control groups of 14.6 μM [10], 68.2 μM [9], and 41.4 μM [12]. The variation of Apo-AI concentrations in the control groups of the different studies are quite large. The Apo-B100 concentrations calculated from the particle concentrations for the control groups are 0.66 μM [10] and 1.28 μM [9]. Compared with 51.9 μM (Apo-A1) and 1.70 μM (Apo-B100) [18] mentioned above it suggests that these values have to be considered with care, even when taking into account that the apolipoprotein concentration determination by immunoassays has an error of about 15% and the control groups are not identical. This means, that one has to be very careful when absolute values of particle concentrations determined by NMR by different programs are essential. However, more important in medical diagnosis are the concentration changes relative to a reference value given by the provider of a test. Indeed, when analyzing the effects of T2D on the lipoprotein subclass concentration changes consistent results are obtained in all studies (see below).

### Variations of apolipoprotein concentrations in impaired glucose tolerance, impaired fasting glucose. and in manifest type 2 diabetes mellitus

Recently, variations of apolipoprotein concentrations in diseases linked to dyslipoproteinemia such as atherosclerosis and coronary heart disease have invoked new interests. The American Societies of Cardiology [36] and the European Society of Cardiology [37] recommend the preferential determination of Apo-B100 concentrations as basic risk assessment for atherosclerosis and coronary heart disease. Mainly the number of apo-B100 particles is predictive for the CHD risk, not the classical cholesterol linked values [38]. The Apo-B100 concentration is also recommended as more suited for therapy control with statins.

In our study, we find a decrease of Apo-B100_NMR_ by -9.8 and -3.2% for fasting and non-fasting conditions when participants with newly detected T2D are compared to the healthy control group. Unfortunately, this decrease is not statistically significant at *P* < 0.05 but for fasting it is significant at an error probability *P* < 0.07. In contrast, in the other studies represented in Fig. 4 an increase of Apo-B100_NMR_ is observed when calculated from the particle concentrations. The simplest explanation for these differences is that this mainly is an effect of the cohort studied. Our data set is compared with data sets containing long-term diabetics together with possible other health problems. For Apo-B48 under normal nutritional conditions a statistically significant increase can be observed for IGT, IGT + IFG, and T2D itself.

### Comparison of the present study with results of similar studies of diabetes related lipoprotein changes

As mentioned in Background there are a number of studies that relate lipoprotein particle concentrations determined by NMR with type 2 diabetes mellitus [7–13]. They differ from each other and from our study as well in many aspects, the number of lipoprotein subclasses and particle sizes defined, the composition of the study cohorts, the fasting state and the method of diagnosis of type 2 diabetes mellitus. Concerning the diagnosis of T2D only Wang et al. [10] used the gold standard for diabetes diagnosis, oGGT, a method that also was used in the present paper. In the other studies the metabolic state of the participants was not unambiguously defined, the diagnosis T2D was not clearly verified and their cohorts may include also subjects with IFG and/or IGT only. The definitions of lipoprotein subclass particle sizes used in our study are given in Table 3. Although the exact definitions differ considerably from study to study, it is possible to find a kind of consensus pattern for the different studies. These definitions are used in Fig. 4 to represent schematically the changes of lipoprotein particle concentrations in T2D described in the present paper and the cited publications. Under standard fasting conditions in all studies the concentration of the smallest HDL particles (HDL A) is significantly increased in T2D, in our case by 5.9%. Under non-fasting condition in our study its increase is even higher, but only statistical significant when people with IFG and/or IGT are included. The particle concentrations of the other HDL subclasses are decreased in most studies. Including the present study (decrease by -18%), the HDL C subclass particle concentrations are significantly decreased in all studies. In our study, the total concentration of HDL particles of all subclasses together (and thus of Apo-A1) is only weakly increased in T2D, it is almost not influenced in our cohort of blood donors (Fig. 4, Table 5). Similar results are also obtained in other studies where Apo-A1 is increased by 0.6% [10] or decreased by -3 or -8% [12, 13].

The concentrations of small LDL particles (LDL A and LDL B) are significantly increased in the five published studies by diabetes (Fig. 4), whereas they are significantly decreased in our study under non-fasting conditions when also the participants with IFG and/or IGT are included. The main difference may be that in our cohort all participants were “healthy” according to the standards defined for blood donors just before diabetes had been detected by oGGT. In our group the average BMI of people with T2D is less than 10% higher than for the control group of healthy volunteers and quite moderate for a group with an average age of 53 and 57 years, respectively (Table 1). This probably means that obesity related dyslipoproteinemia effects are quite small. The concentration of larger LDL particles (LDL C, D, and E) is significantly reduced in three of the five studies and also in our study. VLDL particle concentrations are increased in T2D in all published studies relative to the control group. In our case, only under non-fasting conditions, significance for an increase is reached. The Apo-B100_NMR_ concentration change in subjects with T2D calculated from the particle concentrations varies from + 2.4% [10] to + 26.6% [9]. However, in our case, we find a (not significant) decrease of the Apo-B100 concentration in T2D under fasting and non-fasting conditions (Tables 2 and 4).

Except of our present study, only Wang et al. [10] studied the changes of the chylomicron concentrations in T2D under fasting conditions. They showed a remarkable increase of chylomicron concentrations in diabetic people by 107%. In our case for all three subclasses of chylomicron particles also a significant concentration increase is observed (Fig. 4). Under non-fasting conditions only for large chylomicrons (CM B) a significant concentration increase is observed for people with impaired glucose metabolism. Note that in [10] the nutritional state of the participants was mixed including postprandial data. As discussed above, it is impossible to separate very large VLDLs from small chylomicrons or chylomicron remnants by NMR only, that is the chylomicron fraction may contain a significant contribution of very large VLDL particles in both studies.

Concentrations of chylomicrons and very large VLDLs, respectively, under non-fasting conditions may be the most sensitive marker for T2D in the lipoprofile. This has also proposed by Mora et al. [9] for T2D. Postprandial VLDL and chylomicron concentrations seem also be the most sensitive marker for the risk for cardiovascular diseases [39, 40]. What exactly is happening in type 2 diabetes mellitus pathophysiologically is an open question, is it the lipid resorption, the fatty acid resynthesis, the chylomicron synthesis and clearance or (most likely) all factors together that cause this increase of chylomicrons in blood serum [41, 42]. In T2D insulin does not downregulate the chylomicron synthesis as it does in healthy individuals according to Nogueira et al. [43]. This leads to increased intestinal chylomicron synthesis and secretion in insulin resistance and T2D [44] as also being observed in this study.

## Conclusions

As we have shown the absolute, NMR derived, particle concentrations in many publications cited here vary substantially and the quantifications are probably partly incorrect. Using the temperature dependent visibility/invisibility values from Baumstark et al. [6] as done in the present study gives quite reliable absolute concentrations. In the long-term, a standardization of the method would be required that is based on well-defined size distributions and apolipoprotein concentrations in the different subclasses. The definition of sizes and apolipoprotein stoichiometry should be a future task for the regulatory bodies. However, for the effects of impaired glucose metabolism (IFG, IGT, T2D) on the lipoprotein profile relative concentration changes are mainly important. Here, all studies observe a concentration increase of small HDL particles and a decrease of large HDL particles in T2D (Fig. 4). In addition, an increase of VLDL particle concentrations, and, where data are available, of chylomicrons and chylomicron remnants is observed. Our study is different to most other studies that the diabetic state of our subjects was not known before, our study subjects were healthy according to the criteria set for blood donors (that is also seen in the quite low HbAc1 values) and the diagnosis was based on the gold standard method oGGT. In addition, we can present data for the same subjects under fasting and “normal” nutritional conditions. Here, the diabetic metabolism is easier to observe. Contrary to other studies, in our “healthy” cohort of blood donors the T2D associated dyslipoproteinemia does not significantly change the total concentrations of the lipoproteins produced in the liver under fasting and non-fasting conditions but selectively their subclass distributions. In contrast, under normal nutritional conditions persons with IFG, IGT or T2D show a substantial increase of plasma concentrations of those lipoproteins that are produced in the intestinal tract. An important effect of the insulin resistance gets visible here.

Different to other studies, we observe a slight, significant decrease of the average concentration of small LDL particles (LDL A). This may be again an effect of our cohort of blood donors but one has to be somewhat careful since our cohort itself is not very large.

## Data Availability

Not applicable.

## Abbreviations

IFG: Impaired fasting glucose
IGT: Impaired glucose tolerance
IFG + IGT: IFG combined with IGT
T2D: Type 2 diabetes mellitus

## References

[1] Otvos JD, Jeyarajah EJ, Bennett DW (1991). Quantification of plasma lipoproteins by protein nuclear magnetic resonance spectroscopy. Clin Chem.

[2] Otvos JD (1994). Method and apparatus for measuring classes and subclasses of lipoproteins," Patent US 5343389 A.

[3] Ala-Korpela M, Korhonen A, Keisala J, Horkko S, Korpi P, Ingman LP, Jokisaari J, Savolainen MJ, Kesäniemi YA (1994). 1H NMR-based absolute quantitation of human lipoproteins and their lipid contents directly from plasma. J Lipid Res.

[4] Huber F, Kalbitzer H R, Kremer W. Verfahren zur Bestimmung von Lipoproteinen in Körperflüssigkeiten und Messanordnung dafür. DE 10 2004 026 903 B4 2006.05.18.

[5] Kremer W, Kalbitzer HR, Huber F (2011). Process for the determination of lipoproteins in body fluids. US 7,927,878 B2.

[6] Baumstark D, Kremer W, Boettcher A, Schreier C, Sander P, Schmitz G, Kirchhoefer R, Huber F, Kalbitzer HR (2019). 1H NMR spectroscopy quantifies visibility of lipoproteins, subclasses, and lipids at varied temperatures and pressures. J Lipid Res.

[7] Festa A, Williams K, Hanley AJG, Otvos JD, Goff DC, Wagenknecht LE, Haffner SM (2005). Nuclear magnetic resonance lipoprotein abnormalities in prediabetic subjects in the insulin resistance atherosclerosis study. Circulation.

[8] Hodge AM, Jenkins AJ, English DR, O’Dea K-, Giles GG (2009). NMR-determined lipoprotein subclass profile predicts type 2 diabetes. Diabetes Res Clin Pract.

[9] Mora S, Otvos JD, Rosenson RS, Pradhan A, Buring JE, Ridker PM (2010). Lipoprotein particle size and concentration by nuclear magnetic resonance and incident type 2 diabetes in women. Diabetes.

[10] Wang J, Stančáková A, Soininen P, Kangas AJ, Paananen J, Kuusisto J, Laakso M (2012). Lipoprotein subclass profiles in individuals with varying degrees of glucose tolerance: a population-based study of 9399 Finnish men. J Intern Med.

[11] Mackey RH, Mora S, Bertoni AG, Wassel CL, Carnethon MR, Sibley CT, Goff DC (2015). Lipoprotein particles and incident type 2 diabetes in the multi-ethnic study of atherosclerosis. Diabet Care.

[12] Sokooti S, Szili-Torok T, Flores-Guerrero JL, Osté MCC, Gomes-Neto AW, Kootstra-Ros JE, Heerspink HJL, Connelly MA, Bakker SJL, Dullaart RPF (2020). High-density lipoprotein particles and their relationship to posttransplantation diabetes mellitus in renal transplant recipients. Biomol.

[13] Sokooti S, Flores-Guerrero JL, Kieneker LM, Heerspink HJL, Connelly MA, Bakker SJL, Dullaart RPF (2021). HDL particle subspecies and their association with incident type 2 diabetes: the PREVEND study. J Clin Endocrin Metabol.

[14] Tranaes K, Ding C, Chooi YC, Chan Z, Choo J, Leow MK-S, Magkos F (2021). Dissociation between insulin resistance and abnormalities in lipoprotein particle concentrations and sizes in normal-weight Chinese adults. Front Nutr.

[15] Bergmann A, Li J, Wang L, Schulze J, Bornstein SR, Schwarz PEH (2007). A simplified finnish diabetes risk score to predict type 2 diabetes risk and disease evolution in a German population. Horm Metabol Res.

[16] Martin E, Ruf E, Landgraf R, Hauner, Weinauer F, Martin S (2011). FINDRISK Questionnaire combined with HbA1c testing as a potential screening strategy for undiagnosed diabetes in a healthy population. Horm Metab Res.

[17] Delatour V, Clouet-Foraison N, Gaie-Levrel F, Marcovina SM, Hoofnagle, Kuklenyik Z, Caulfield MP, Otvos JD, Krauss RM, Kulkarni KR, Contois JH, Remaley AT, Vesper HW, Cobbaert CM, Giller P (2018). Comparability of Lipoprotein Particle Number Concentrations Across ES-DMA, NMR, LC-MS/MS, immunonephelometry, and VAP: In Search of a candidate reference measurement procedure for apoB and non-HDL-P standardization. Clin Chem.

[18] Monsonis-Centelles S, Hoefsloot HCJ, Engelsen SB, Smilde AK, Lind MV (2020). Repeatability and reproducibility of lipoprotein particle profile measurements in plasma samples by ultracentrifugation. Clin Chem Lab Med.

[19] Matsuda D, Nishida M, Arai T, Hanada H, Yoshida H, Yamauchi-Takihara K, Moriyama T, Tad N, Yamashita S (2014). Reference interval for apolipoprotein B-48 concentration in healthy Japanese Individuals. J Atheroscler Thromb.

[20] Phillips ML, Pullinger C, Kroes I, Kroes J, Hardman DA, Chen G, Curtiss LK, Gutierrez MM, Kane JP, Schumaker VN. A single copy of apolipoprotein B-48 is present on the human chylomicron remnant. J Lipid Res 1997;38:1170–7. 9215545

[21] Wiklund O, Dyer CA, Tsaoe BP, Curtiss LK (1985). Stoichiometric binding of apolipoprotein specific monoclonal antibodies to low density lipoproteins. J Biol Chem.

[22] Matera R, Horvath KV, Nair H, Schaefer EJ, Asztalos BF (2018). HDL Particle measurement: comparison of 5 methods. Clin Chem.

[23] He Y, Song HD, Anantharamaiah GM, Palgunachari MN, Bornfeldt KE, Segrest JP, Heinecke JW (2019). Apolipoprotein A1 Forms 5/5 and 5/4 antiparallel dimers in human high-density lipoprotein. Mol Cell Proteomics.

[24] Fang Y, Gursky O, Atkinson D (2003). Lipid-binding studies of human apolipoprotein A-I and its terminally truncated mutants. Biochemistry.

[25] Bibow S, Polyhach Y, Eichmann C, Chi CN, Kowal J, Albiez S, McLeod RA, Stahlberg H, Jeschke G, Günter P, Riek R (2017). Solution structure of discoidal high-density lipoprotein particles with a shortened apolipoprotein A-I. Mature. Struct Mol Biol.

[26] Brouillette CG, Anantharamaiah GM, Engler JA, Borhani DW (2001). Structural models of human apolipoprotein A-I: a critical analysis and review. Biochim Biophys A.

[27] Hutchins PM, Ronsein GE, Monette JS, Pamir N, Wimberger J, He Y, Anantharamaiah GM, Kim DS, Ranchalis JE, Jarvik GP, Vaisar T, Heinecke JW (2014). Accurate quantification of high density lipoprotein particle concentration by calibrated ion mobility analysis. Clin Chem.

[28] Tian J, Chen H, Liu P, Wang C, Chen Y (2019). Fasting apolipoprotein B48 is associated with large artery atherosclerotic stroke: a case control study. Sci Rep.

[29] Konishi T, Fujiwara R, Saito T, Satou N, Hayashi Y, Crofts N, Iwasaki I, Abe Y, Kawata S, Shikawa T (2022). Human lipoproteins comprise at least 12 different classes that are lognormally distributed. PLoS ONE.

[30] Nakajima K, Nakano T, Tokita Y, Nagamine T, Inazu A, Kobayashi J, Mabuchi H, Kimber L, Stanhope KL, Havel PJ, Okazaki M, Ai M, Tanaka A (2011). Postprandial lipoprotein metabolism VLDL vs chylomicrons. Clin Chem A.

[31] Kronenberg F, Lobentam EV, Konig P, Utermann G, Dieplinger H (1994). Effect of sample storage on the measurement of lipoprotein[a], apolipoproteins B and A-IV, total and high density lipoprotein cholesterol and triglycerides. J Lipid Res.

[32] Zivkovic AM, Wiest MM, Nguyen UT, Davis R, Watkins SM, German JB (2009). Effects of sample handling and storage on quantitative lipid analysis in human serum. Metabolomics.

[33] Cuhadar S, Koseoglu M, Atay A, Dirican A (2013). The effect of storage time and freeze-thaw cycles on the stability of serum samples. Biochem Med.

[34] Loo RL, Lodge S, Kimhofer T, Bong SH, Begum S, Whiley L, Gray NC, Lindon JC, Nitschke P, Lawler NG, Schafer H, Spraul M, Richards T, Nicholson JK, Holmes E (2020). Quantitative in-vitro diagnostic NMR spectroscopy for lipoprotein and metabolite measurements in plasma and serum: recommendations for analytical artifact minimization with special reference to COVID-19/SARS-CoV-2 Samples. J Proteome Res.

[35] Kaess B, Fischer M, Baessler A, Stark K, Huber F, Kremer W, Kalbitzer HR, Schunkert H, Riegger G, Hengstenberg C (2008). (2008) The lipoprotein subfraction profile: heritability and identification of quantitative trait loci. J Lipid Res.

[36] Grundy SM, Stone NJ, Bailey AL, Beam C, Birtcher KK, Blumenthal RS, Braun LT, de Ferranti S, Faiella-Tommasino J, Forman DE, Goldberg R, Eidenreich PA, Hlatky MA, Jones DW, Lloyd-Jones D, Lopez-Pajares N, Ndumele CF, Orringer CE, Peralta CA, Saseen JJ, Smith SC, Sperling L, Virani DS, Yeboah J (2019). AHA/ACC/AACVPR/AAPA/ABC/ACPM/ADA/AGS/ APhA/ASPC/NLA/PCNA guideline on the management of blood cholesterol: executive summary. J Am Coll Cardiol.

[37] Mach F, Baigent C, Catapano AL, Koskinas KC, Casula M, Badimon L, Chapman MJ, De Backer GG, Delgado V, Ference BA, Graham IM (Ireland), Halliday A, Landmesser U, Mihaylova B, Pedersen TR, Riccardi G, Richter DJ, Sabatine MS, Taskinen MR, Tokgozoglu L, Wiklund O. 2019 ESC/EAS Guidelines for the management of dyslipidaemias: lipid modification to reduce cardiovascular risk. Eur Heart J. 2020;41:111-188. 10.1093/eurheartj/ehz455 31504418

[38] Kohli-Lyncha CN, Thanassoulisc G, Morana AE, Sniderman AD (2020). The clinical utility of apoB versus LDL-C/non-HDL-C. Clin Chim A.

[39] Mora S, Rifai N, Buring JE, Ridker PM (2008). Fasting compared with nonfasting lipids and apolipoproteins for predicting incident cardiovascular events. Circulation.

[40] Bansal S, Buring JE, Rifai N, Mora S, Sacks FM, Ridker PM (2007). Fasting compared with nonfasting triglycerides and risk of cardiovascular events in women. JAMA.

[41] Haas ME, Attie AD, Biddinger SB (2013). The regulation of ApoB metabolism by insulin. Trends Endocrinol Metabol.

[42] Giammanco A, Cefalù AB, Noto D, Averna MR (2015). The pathophysiology of intestinal lipoprotein production. Front Physiol.

[43] Nogueira J-P, Maraninchi M, Béliard S, Padilla N, Duvillard L, Mancini J, Nicolay A, Xiao C, Vialettes B, Lewis GF, Valéro R (2012). Absence of acute inhibitory effect of insulin on chylomicron production in type 2 diabetes. Arterioscler Thromb Vasc Biol.

[44] Stahel P, Xiao C, Nahmias A, Lewis GF (2020). (2020) Role of the Gut in Diabetic Dyslipidemia. Froniers Endocrinol.

